# Adherence to the Mediterranean Diet, Dietary Patterns and Body Composition in Women with Polycystic Ovary Syndrome (PCOS)

**DOI:** 10.3390/nu11102278

**Published:** 2019-09-23

**Authors:** Luigi Barrea, Angela Arnone, Giuseppe Annunziata, Giovanna Muscogiuri, Daniela Laudisio, Ciro Salzano, Gabriella Pugliese, Annamaria Colao, Silvia Savastano

**Affiliations:** 1 Dipartimento di Medicina Clinica e Chirurgia, Unit of Endocrinology, Federico II University Medical School of Naples, Via Sergio Pansini 5, 80131 Naples, Italy; angela.arnone15@gmail.com (A.A.); giovanna.muscogiuri@gmail.com (G.M.); daniela.laudisio@libero.it (D.L.); cirosalzano89@gmail.com (C.S.); robiniapugliese@gmail.com (G.P.); colao@unina.it (A.C.); sisavast@unina.it (S.S.); 2 Department of Pharmacy, University of Naples “Federico II”, Via Domenico Montesano, 49, 80131 Naples, Italy; giuseppe.annunziata@unina.it

**Keywords:** Mediterranean Diet, Dietary pattern, Body Composition, Phase Angle, Polycystic Ovary Syndrome (PCOS), Nutritionist

## Abstract

Polycystic ovary syndrome (PCOS) is the most common female endocrine disorder and is characterized by chronic anovulation, hyperandrogenism, and polycystic ovaries. Obesity, low-grade chronic inflammatory status, and insulin-resistance (IR) often coexist in PCOS. The Mediterranean diet (MD) is an anti-inflammatory dietary pattern, which is rich in complex carbohydrates and fiber, and high in monounsaturated fat. There is a close association among obesity, low-grade chronic inflammation, IR, and hormonal derangements in PCOS. The main aim of the present study was to evaluate the adherence to MD, the dietary intake, and the body composition and their association with PCOS clinical severity in a cohort of treatment-naïve women with PCOS when compared with a control group of healthy women matched for age and body mass index (BMI). In this case-controlled, cross-sectional study, we enrolled 112 patients with PCOS and 112 controls. PREvención con DIetaMEDiterránea (PREDIMED) and seven-day food records were used to evaluate the degree of adherence to the MD and dietary pattern, respectively. Body composition was evaluated by bioelectrical impedance analysis (BIA) phase-sensitive system. Testosterone levels and Ferriman-Gallwey score assessed the clinical severity of PCOS. C-reactive protein (CRP) levels were determined with a nephelometric assay with CardioPhase high sensitivity. PCOS women showed higher testosterone levels, Ferriman-Gallwey score, fasting insulin and glucose levels, and Homeostatic Model Assessment (HoMA)-IR when compared with the control group (*p* < 0.001). In addition, we found that the PCOS women consumed less extra-virgin olive oil, legumes, fish/seafood, and nuts compared with control group. Despite no differences in energy intake between the two groups, the PCOS women consumed a lower quantity of complex carbohydrate, fiber, monounsaturated fatty acids (MUFA), and n-3 polyunsaturated fatty acid (PUFA), and higher quantity of simple carbohydrate, total fat, saturated fatty acid (SFA), PUFA and n-6 PUFA than the control group. The PCOS women have an adverse body composition when compared with controls, with the lowest values of phase angle (PhA) and fat-free mass (*p* < 0.001). Additionally, after adjusting for BMI and total energy intake, testosterone levels showed significant negative correlations with PREDIMED score (*p* < 0.001) and consumption of protein (*p* = 0.005), complex carbohydrate (*p* < 0.001), fiber (*p* < 0.001), MUFA (*p* < 0.001), n-3 PUFA (*p* = 0.001), and positive associations with CRP levels, simple carbohydrate, SFA, n-6 PUFA (*p* < 0.001, respectively), and PUFA (*p* = 0.002). The cut-off for PREDIMED score ≤ 6 (*p* < 0.001, area under the curve (AUC) 0.848, standard error 0.036, 95% confidence interval (CI) 0.768 to 0.909) could serve as a threshold for significantly increased risk of high value of testosterone levels. In conclusion, a novel direct association between the adherence to MD and the clinical severity of the disease was reported in women with PCOS. This association could support a therapeutic role of foods and nutrients of the Mediterranean dietary pattern in the PCOS pathogenesis likely involving their inflammatory status, IR, and hyperandrogenemia. In addition, we reported a different body composition that is characterized by lower PhA and fat-free mass than controls. These data suggested a role of PhA as a useful marker of the clinical severity of this syndrome and provided strong evidence regarding the strategic relevance of the nutritional assessment in the management of women with PCOS.

## 1. Introduction

Polycystic ovary syndrome (PCOS) is a multifactorial endocrine metabolic disorder [1] that affects up to 18% of women of reproductive age [2]. PCOS is characterised by a spectrum of different phenotypes and it is diagnosed when at least two of the three Rotterdam criteria are present: (i) clinical hyperandrogenism (with hirsutism, acne, seborrhea, and alopecia) and/or with high circulating androgens levels; (ii) presence of ovarian cysts assessed by ultrasound examination; and, (iii) oligo-amenorrhea with oligo-anovulation [3]. In clinical practice, the Ferriman-Gallwey score is an efficient tool for assessing hirsutism, which in turn is commonly correlated with biochemical hyperandrogenism [4]. Beyond hormonal derangements, obesity, insulin-resistance (IR), with associated compensatory hyperinsulinemia [5], and a low-grade chronic inflammatory status often coexist with PCOS [6]. Although several studies have been carried out to investigate the association of metabolic alterations and PCOS, it is still unknown why obesity is often a common finding in women with PCOS. In particular, obesity and obesity-related low-grade inflammation both contribute to the onset of IR, by amplifying the metabolic and reproductive outcomes [7]. IR and the compensatory hyperinsulinemia might empower the androgenic activity, while the high levels of androgens may further blunt IR through alterations of the body composition [8], which results in a vicious circle that leads to a general worsening of this pathological status. Metabolic and endocrine derangements of PCOS both contribute to making women with PCOS more prone to develop metabolic syndrome, type 2 diabetes mellitus, and infertility [9,10,11]. Since obesity worsens the clinical presentation of PCOS, according to the Recommendations from the International Evidence-based Guideline for the Assessment and Management of PCOS, weight management is one of the main treatment strategies [12]. However, it is still debated what is the best effective nutritional pattern that should be followed to lose weight in PCOS. Among the different nutritional strategies, the Mediterranean diet (MD) is commonly recognised as a health-promoting dietary pattern due to its peculiar features, including the regular consumption of unsaturated fats, low glycaemic index carbohydrates, fiber, vitamins and antioxidants, and moderate amount of animal-derived proteins [13]. In addition to weight loss, the MD has been reported to have a well-established anti-inflammatory activity, which is mainly due to the microbiota-derived production of short chain fatty acids that are induced by dietary fibre [14] and the high intake of both polyunsaturated fatty acids (PUFA) omega 3 and antioxidants contained in fruits, vegetables, extra-virgin olive oil, and wine [15,16,17]. However, no evidence is available in the literature regarding to the association between the adherence to MD and the PCOS severity.

Beyond the MD, to date, few data are available on the dietary intake in women with PCOS by using the seven-day food records, which is recognised as the “gold standard” for careful nutritional assessments [18,19]. Douglas and colleagues (2006) estimated the dietary intake in women with PCOS by using both a multiple-choice food questionnaire and a four-day food record and observed that, although the intake of total energy and macro- and micronutrients were similar, PCOS women had a significantly higher intake of high glycaemic index foods (mainly white bread and fried potatoes) than age- and body mass index (BMI)-matched controls [20]. 

In clinical practice, body composition is commonly measured by bioelectrical impedance analysis (BIA), which is a non-invasive method for evaluation of body composition with a high agreement with Dual-energy X-ray absorptiometry [21]. Phase angle (PhA) is a BIA-derived measure that is associated with the inflammatory status. Studies investigating body composition by BIA reported that in PCOS women higher metabolic dysfunction was associated with higher fat mass (FM) and fat-to-lean mass ratio as compared to controls [22]. In addition, body fat percentage was positively associated with levels of inflammatory biomarkers [23].

In this context, the main aim of the present study was to evaluate the adherence to MD, the dietary intake assessed by seven-day food records, and the body composition assessed by BIA and their association with PCOS clinical severity in a cohort of treatment-naïve women with PCOS as compared with a control group of healthy women matched for age and BMI. When considering the coexistence of both inflammation and obesity, and while taking into account the well-known anti-inflammatory effects of MD, we suppose that PCOS women should be more prone to follow an unhealthy diet, poorly adherent to the Mediterranean style, which contributes to worsening their condition; furthermore, since various studies demonstrated the association between PhA and inflammation, we hypothesize to find this relationship also in PCOS patients.

## 2. Materials and Methods 

### 2.1. Design and Setting

This cross-sectional, observational study was carried out in patients with PCOS attending the Unit of Endocrinology, Department of Clinical Medicine and Surgery, University Federico II of Naples (Italy), from January 2014 to January 2019. The study has been approved by the Local Ethical Committee (n. 05/14) and carried out in accordance with the Code of Ethics of the World Medical Association (Declaration of Helsinki) for experiments that involved humans. The aim of the study was clearly explained to all of the study participants and a written informed consent was obtained. 

### 2.2. Population Study

The subjects were enrolled at the outpatient Endocrinology clinic. The study included 112 treatment-naïve women that were affected by PCOS attending the Outpatient Clinic of the Unit of Endocrinology in our Department. 

One hundred-twelve Caucasian healthy subjects (ascertained by participant questionnaire excluding the presence of clinical conditions that potentially influences fluid balance, i.e., myocardial, renal, or endocrine diseases), age, and BMI matched were chosen as the controls among hospital volunteers and employees from the same geographical area around Naples (Italy). To increase the homogeneity of the patient sample, we only included treatment-naïve women. 

Eligible patients were those with a diagnosis of PCOS classified by the European Society for Human Reproduction and Embryology/American Society for Reproductive Medicine (ESHRE/ASRM) diagnosis [3]. This includes the presence of two of the three features of hyperandrogenism (either clinical (hirsutism by elevated Ferriman-Gallwey score) or biochemical (elevated testosterone or free androgen index), oligomenorrhea (interval between two menstrual periods more than 35 days), or amenorrhea (no vaginal bleeding for at least six months) and the presence of polycystic ovaries on ultrasound scan (≥12 follicles measuring 2–9 mm in diameter, or ovarian volume >10 mL in at least one ovary).

The inclusion criteria for all groups were: premenopausal women who were normal-weight, overweight or obese (until BMI 39.9 kg/m^2^), aged 18–40 years, a lack of underlying metabolic disease (type 2 diabetes, hypertension, diagnosed anemia, or any other metabolic disease requiring a special diet).

The exclusion criteria for all groups were the following:

1. Age < 18 years and > 40 years;
2. Menopause (defined as amenorrhea for ≥3 years or amenorrhea for ≥1 but <3 years and plasma follicle-stimulating hormone concentrations elevated to the postmenopausal range); pregnancy or lactation in the past 6 months;
3. Hyperandrogenism and/or biochemical hyperandrogenemia, oligomenorrhea due to secondary etiologies as per the Endocrine Society Clinical Practice Guidelines and previous publications including endocrine disorders (congenital adrenal hyperplasia, androgen-secreting tumors, Cushing’s syndrome, hyperprolactinaemia, thyroid dysfunction and adrenal disorders) [24];
4. Pre-existing systemic or psychiatric disease and use of medications that impact carbohydrate or lipid metabolism (oral contraceptive pills, metformin, anti-epileptics, anti-psychotics, statins and fish oil);
5. Specific nutritional regimens or hypocaloric diet in the last three months, including vegan or vegetarian diets; supplementation with antioxidants, vitamins or minerals;
6. Occasional or current of use of drugs that could influence fluid balance, including non-steroidal anti-inflammatory drugs, diuretics, laxative use; and,
7. Women patients with implanted pacemakers or defibrillators because of the theoretical possibility of interference with the device activity due to the field of current induced by the impedance measurements.

The clinical and biochemical assessment were done between 8 am and 12 am, after an overnight fast.

### 2.3. Sample Size Justification and Power

The sample size was calculated on the basis of a previous study that reported prevalence of PCOS as 12% in women of reproductive age [25]. The calculation of the sample size was performed by considering two independent study groups; the effect size 0.95 with type I error of 0.05 and a power of 95%. The number of subjects to be enrolled was found to be 99 per group that we decided to round up to 112 with a total of 224 total subjects being enrolled in the study to replace drop out patients. 

The power was calculated by the differences of means ± standard deviation (SD) of PREvención con DIetaMEDiterránea (PREDIMED) score in the PCOS patients and control group (6.97 ± 2.72 vs. 8.12 ± 2.80, respectively). When considering the number of cases required in each group of 100, were set at 112 of group, a type I (alpha) error of 0.05 (85%), and a type II (beta) of 0.05, the calculated power size was 85%. The calculation of sample size and power were performed while using Sample Size Calculator Clinical Calc (https://clincalc.com/stats/samplesize.aspx).

### 2.4. Lifestyle Habits and Anthropometric Measurements 

We defined smoking at least one cigarette per day as current smokers subjects, former smokers were subjects who stopped smoking at least one year before the interview, and non-current smokers the remaining participants. The participants habitually engaged in at least 30 min/day of aerobic exercise (YES/NO) were defined as physically active, as we have already fully reported in previous studies [26,27,28]. 

Measurements were performed between 8 am and 12 pm. All of the subjects were measured after an overnight fast. The anthropometric measurements were performed by the same operator (a nutritionist experienced in providing nutritional assessment and body composition), according to the International Society for the Advancement of Kinanthropometry (ISAK 2006). 

All of the anthropometric measurements were taken with subjects only wearing light clothes and without shoes, as previously reported [27,29,30,31]. In each subject, weight and height were measured to calculate the BMI (weight (kg) divided by height squared (m^2^), kg/m^2^). Height was measured to the nearest 0.5 cm while using a wall-mounted stadiometer (Seca 711; Seca, Hamburg, Germany). Body weight was determined to the nearest 0.1 kg while using a calibrated balance beam scale (Seca 711; Seca, Hamburg, Germany). BMI was classified according to World Health Organization’s criteria with normal weight: 18.5–24.9 kg/m^2^; overweight, 25.0–29.9 kg/m^2^; grade I obesity, 30.0–34.9 kg/m^2^; grade II obesity, 35.0–39.9 kg/m^2^ [32].

Waist circumference (WC) was measured to the closest 0.1 cm while using a non-stretchable measuring tape at the natural indentation or at a midway level between lower edge of the rib cage and iliac crest if no natural indentation was visible, in according to the National Center for Health Statistics [33].

### 2.5. Nutritional Assessments 

The adherence to the MD was assessed using PREDIMED questionnaire, consisting of 14 items [34]. A qualified nutritionist administered this questionnaire, which has already been used in previous studies [35,36,37,38], during a face-to-face interview. Briefly, by assigning a score 1 and 0 for each items, PREDIMED score was calculated, as follows: 0–5, lowest adherence; score 6–9, average adherence; score ≥10, highest adherence [34].

As we have already fully reported in previous studies [39,40,41,42], the dietary assessments were obtained by a face-to-face interview that a qualified nutritionist administered. In detail, a photographic food atlas (≈1000 photographs) of known portion sizes was used to quantify foods and drinks [43] and the seven-day food records were used to collect dietary data, including beverage intakes. On the basis of these records, the nutritionist calculated the total energy intake and the quantities of macronutrients.

### 2.6. Body Composition

Body composition was assessed while using a BIA phase-sensitive system by experienced observers (an 800-µA current at a frequency single-frequency of 50 kHz BIA 101 RJL, Akern Bioresearch, Florence, Italy) [44], as previously reported [45,46,47,48]. The exam was performed as suggested by the European Society of Parental and Enteral Nutrition (ESPEN) [49]. The electrodes were placed on the hand and the ipsilateral foot, according to Kushner [50]. PhA is calculated as the relationship between the resistance (R) of tissues, which is mainly dependent on tissue hydration and their reactance (Xc), which is associated with cellularity, cell size, and integrity of the cell membrane [51,52,53,54], according to the following formula: PhA (°, degrees) = Xc/R * (180/π). The same operator and the same device obtained BIA determinations under strictly standardized conditions in order to avoid interobserver and inter-device variability. The BIA was routinely checked with resistors and capacitors of known values. Reliability for within-day and between-day measurements were <1.9% for R, <2.1% for Xc, and <2.8% for R, <2.3% for Xc, respectively. The coefficient of variation (CV) of repeated measurements of R and Xc at 50 kHz was assessed in 20 females (10 PCOS and 10 controls): CVs were 1.6% for R and 1.8% for Xc. 

### 2.7. Assay Methods

The samples were collected in the morning between 8 and 10 am, after an overnight fast of at least 8 h and stored at −80 °C until being processed. Fasting plasma glucose was analyzed with a Roche Modular Analytics System in the Central Biochemistry Laboratory of our Institution. The fasting insulin levels were measured by a solid-phase chemiluminescent enzyme immunoassay using commercially available kits (Immulite Diagnostic Products Co., Los Angeles, CA, USA). Homeostasis model assessment (HoMA)-IR was calculated according to Matthews et al. [55]. A value of HoMA-IR >2.5 was used as cut-off of IR. C-Reactive Protein (CRP) levels were determined with a nephelometric assay with CardioPhase high sensitivity from Siemens Healthcare Diagnostics (Marburg, Germany). Serum testosterone was measured by chemiluminescent enzyme immunoassay (Immulite 2000, Diagnostic Products Corp.). The intra- and inter-assay CV were <7% for all of the assays performed.

### 2.8. Clinical Hyperandrogenism

The degree of hirsutism was evaluated, as originally described by Ferriman-Gallwey hirsutism scoring scale, which measures nine androgen sensitive areas in the body [56]. A single Endocrinologist performed the scoring assessment of each patient. Ferriman-Gallwey score evaluates 11 different body parts, with scores ranging from 0 (no excessive terminal hair growth visible) to four (extensive hair growth visible) for each body part evaluated. A maximum score of 36 is possible, but a score of >7 or more was considered to be diagnostic of hirsutism, as defined by the 95th percentile of data initially collected by Ferriman [57].

### 2.9. Statistical Analysis

The data distribution was evaluated by Kolmogorov-Smirnov test and data not normally distributed were normalized by logarithm. Skewed variables (insulin, HoMA-IR, CRP levels, testosterone, total carbohydrate intake, n-6 PUFA intake) were back-transformed for presentation in tables and figures. The results are expressed as mean ±SD. The chi square (χ^2^) test was used to determine the significance of differences in frequency distribution of smoking habit, physical activity, BMI categories, WC cut-offs, adherence to the MD, and dietary components included in the PREDIMED questionnaire. 

Differences between PCOS patients and control group were analysed by Student’s independent t-test, while the differences among the several parameters with the PREDIMED categories and CRP levels, HoMA-IR, testosterone levels, and Ferriman-Gallwey score were analysed by between-groups ANOVA test followed by the Bonferroni post-hoc test. The correlations between study variables were performed while using Pearson r correlation coefficients after adjusting for BMI and total energy intake. In addition, a multiple linear regression analysis model (stepwise method), expressed as R^2^, Beta (β) and t, with testosterone levels as the dependent variable, was used to estimate the predictive value of a food items, WC, PREDIMED score, CRP levels, HoMA-IR, and nutrient intake (protein, complex carbohydrate, simple carbohydrate, saturated fat acid (SFA), monounsaturated fat acid (MUFA), PUFA, n-6 PUFA, n-3 PUFA, fiber). Receiver operator characteristic (ROC) curve analysis were performed to establish sensitivity and specificity, area under the curve (AUC), and confidence interval (IC), as well as cut-off values for PREDIMED score in detecting testosterone levels that were above the median values in the PCOS women. Test AUC for ROC analysis was also calculated and we entered 0.848 for AUC ROC and 0.5 for null hypothesis values. An α level of 0.05 (type 1 error) and a β level of 0.20 (type II error) were used as the cut-off values for statistical significance. Only variables that had a *p*-value < 0.05 in the univariate analysis (partial correlation) were entered. Variables with a variance inflation factor (VIF) >10 were not considered to avoid multicollinearity. Values ≤ 5% were considered to be statistically significant. Data were collected and analysed while using the MedCalc® package (Version 12.3.0 1993–2012–Mariakerke, Belgium). 

## 3. Results

The study population consisted of 224 participants, 112 patients with PCOS, and 112 women as the control group. All of the cases and controls completed the study protocol, including the nutritional assessment, the PREDIMED questionnaire, the seven-days food diary records, and BIA measurements.

Table 1 reports the lifestyle habits, anthropometric measurements, adherence to the MD, CRP levels, hormonal, and biochemical parameters, the presence of clinical signs, and symptoms of hyperandrogenism, and/or biochemical hyperandrogenemia of the cases and the controls matched for age and BMI. As expected, the PCOS women showed higher testosterone levels, Ferriman-Gallwey score, fasting insulin and glucose levels, and HoMA-IR when compared with the control group (*p* < 0.001). Among the PCOS women, there were no differences in their smoking habits, physical activity, and BMI categories, whereas more PCOS women presented WC >cut-off (*p* < 0.001) and a lower adherence to the MD than controls (*p* < 0.001).

Analysing the response frequency of dietary components included in the PREDIMED questionnaire in detail, we found that the PCOS women consumed less extra-virgin olive oil, legumes, fish/seafood, and nuts, as compared with the control group (Table 2). 

Data on Mediterranean food frequencies were further analysed by using the 7-day food records. As shown in the Table 3, in spite of no differences in energy intake between the two groups, the PCOS women consumed a lower quantity of complex carbohydrate, fiber, unsaturated fatty acids, MUFA, and n-3 PUFA, and higher quantity of simple carbohydrate, total fat, SFA, PUFA, and n-6 PUFA than the control group. The body composition that was assessed by BIA parameters of the PCOS women and the control group are shown in Table 3. In particular, the PCOS women have lower values of Xc (*p* = 0.028), PhA (*p* < 0.001), fat-free mass (FFM) (*p* < 0.001), total body water (TBW) (*p* = 0.001), and intracellular water (ICW) (*p* = 0.004), and higher values of FM (*p* < 0.001) and extracellular water (ECW) (*p* = 0.004) than the controls. 

The PCOS women were examined by stratifying above and below the median of testosterone (22.27 ng/dL), (Table 4). The PCOS women that were above the median of testosterone had the worse anthropometric measurements, adherence to the MD, CRP levels, hormonal and biochemical parameters, dietary pattern, and body composition. 

**Figure 1:**
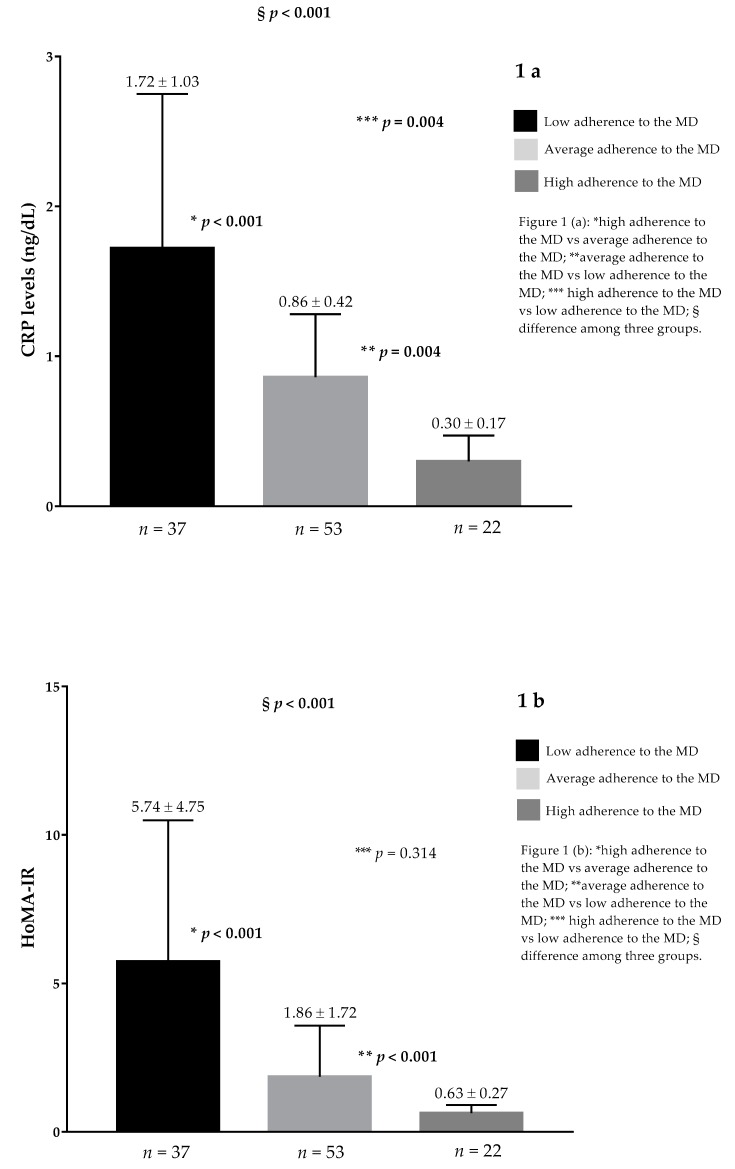

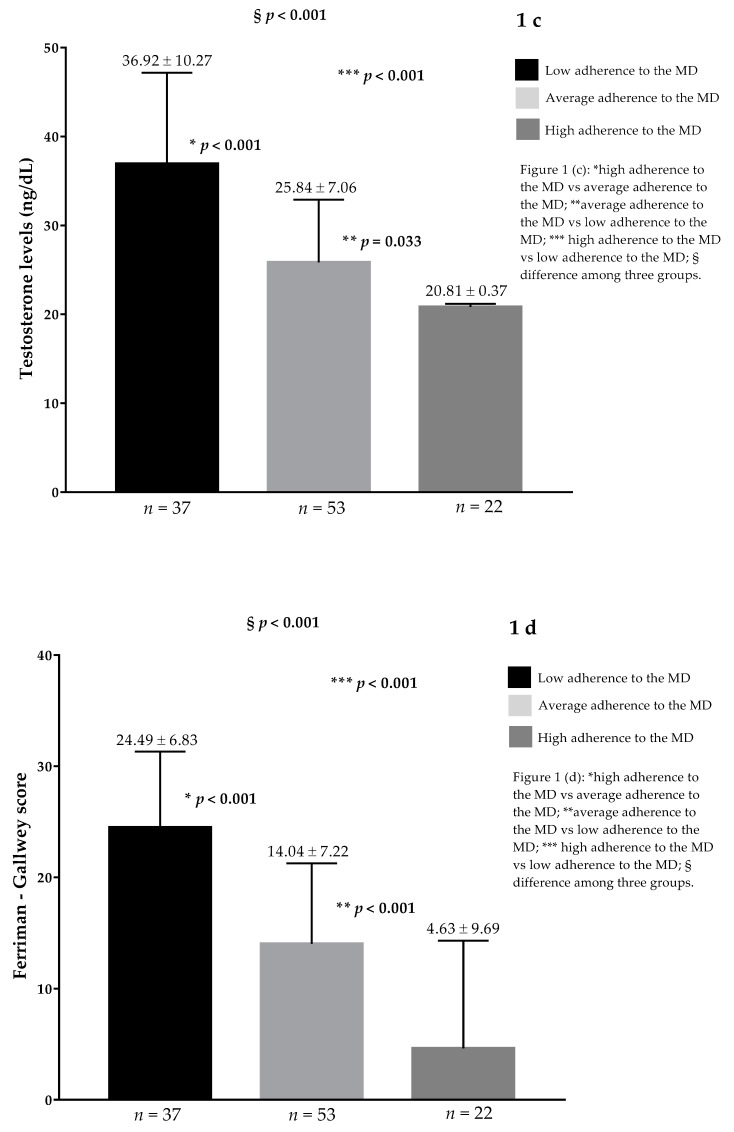
In Figure 1 were reported the differences of CRP levels, HoMA-IR, testosterone levels, and Ferriman-Gallwey score of the PCOS women on the basis of the PREDIMED categories. As showed in Figure 1, the PCOS women with a low adherence to the MD presented higher CRP levels (**a**), HoMA-IR (**b**), testosterone levels (**c**), and Ferriman-Gallwey score (**d**) than average and high adherers to the MD (*p* < 0.001, respectively). CRP, C-reactive protein; MD, Mediterranean diet; HoMA-IR, Homeostasis model assessment insulin resistance; PCOS, Polycystic ovary syndrome; PREDIMED, PREvención con DIetaMEDiterránea.

In Figure 1 are reported the differences of CRP levels, HoMA-IR, testosterone levels, and Ferriman-Gallwey score of the PCOS women on the basis of the PREDIMED categories. As shown in Figure 1, the PCOS women with a low adherence to the MD presented higher CRP levels (Figure 1a), HoMA-IR (Figure 1b), testosterone levels (Figure 1c), and Ferriman-Gallwey score (Figure 1d) than the average and high adherers to the MD (*p* < 0.001, respectively). 

### Correlation Studies

The correlations between testosterone levels with anthropometric parameters, adherence to the MD, CRP levels, dietary intake, and body composition of the PCOS women are summarized in Table 5. Additionally, after adjusting for BMI, and total energy intake, testosterone levels showed significant negative correlations with PREDIMED score (*p* < 0.001) and consumption of protein (*p* = 0.005), complex carbohydrate (*p* < 0.001), fiber (*p* < 0.001), MUFA (*p* < 0.001), n-3 PUFA (*p* = 0.001), and positive associations with WC, CRP levels, simple carbohydrate, SFA, n-6 PUFA (*p* < 0.001, respectively), and PUFA (*p* = 0.002). 

As expected, CRP levels were negatively correlated with PREDIMED score and PhA (r = −0.717, *p* < 0.001 and r = −0.493, *p* < 0.001), however, while the correlation with PhA was lost after adjusting for BMI and total energy intake (r = −0.151, *p* = 0.114), the correlation with PREDIMED score and CRP levels remained significant, even after adjusting for these potential confounders (r = −0.325, *p* = 0.001).

To assess the relative prognostic value of the WC, the adherence to the MD, CRP levels, HoMA-IR, and daily energy and nutrients intake (protein, complex carbohydrate, simple carbohydrate, SFA, MUFA, PUFA, n-6 PUFA, n-3 PUFA, and fiber) to predict the clinical severity of PCOS, we performed a multiple linear regression analysis model, including these parameters. While using this model, the CRP levels entered at the first step (*p* < 0.001), followed by PREDIMED score (*p* < 0.001) and MUFA intake (*p* = 0.001). As reported in Table 6, the model explained a total of 73.8% of the total variability, including all three independent variables into the multiple regression.

**Figure 2:**
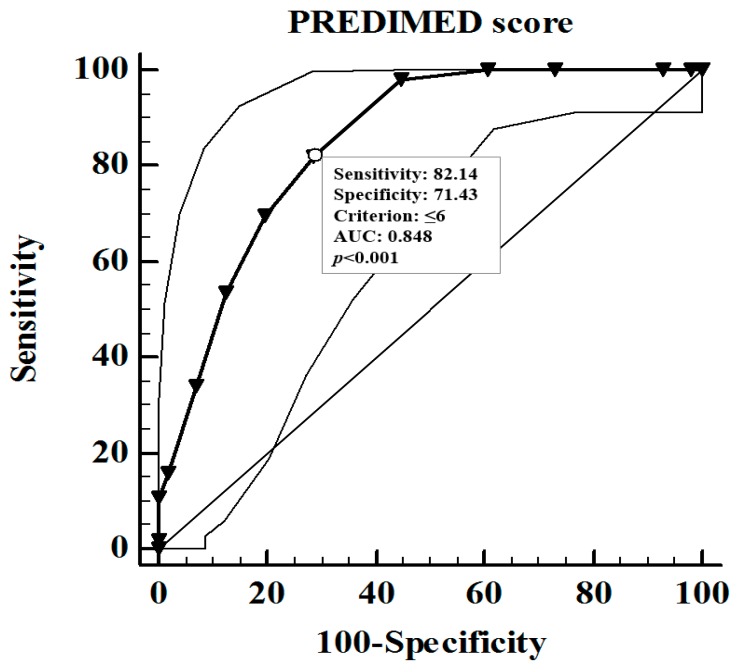
The ROC analysis was performed to determine the cut off values of the PREDIMED score that was predictive of highest values of testosterone levels (above the median value 22.27 ng/dL). A value of PREDIMED score of ≤ 6 (*p* < 0.001, AUC 0.848, standard error 0.036, 95% CI 0.768 to 0.909) could serve as a threshold for a significantly increased risk of high value of testosterone levels. PREDIMED, PREvención con DIetaMEDiterránea; ROC, Receiver operator characteristic; AUC, area under the curve; CI, confidence interval.

The ROC analysis was performed to determine the cut off values of the PREDIMED score that was predictive of the highest values of testosterone levels (above the median value 22.27 ng/dL) (Figure 2). A value of PREDIMED score of ≤ 6 (*p* < 0.001, AUC 0.848, standard error 0.036, 95% CI 0.768 to 0.909) could serve as a threshold for a significantly increased risk of high value of testosterone levels.

## 4. Discussion

In this cross-sectional, observational study, we investigated the adherence to the MD, the dietary intake assessed by seven-day food records, and the body composition assessed by BIA and their associations with testosterone levels as index of PCOS clinical severity in a cohort of treatment-naïve women with PCOS when compared with a control group of healthy women matched for age and BMI. In particular, our results showed that the PCOS women had lower adherence to the MD and consumed less extra-virgin olive oil, legumes, fish, and nuts than the controls. To the best of our knowledge, this is the first study that investigated the adherence to the MD in PCOS women and the intake of single nutrients.

In particular, the analysis of the seven-day food records showed that the PCOS women had a different dietary pattern, with a higher consumption of simple carbohydrates and SFA, and low consumption of complex carbohydrates, fiber, and MUFA as compared to controls. Of interest, this unhealthy dietary pattern was associated with more severe hyperandrogenemia, inflammatory status and IR. In particular, the inflammatory status, the adherence to the MD, and MUFA intake were the factors that exerted the most powerful influence on testosterone levels. In addition, a PREDIMED score ≥6 was found as the most sensitive and specific cut-point to predict value of testosterone levels above the median values. 

The MD is a well-established health-promoting dietary pattern. In particular, there is evidence that the adherence to the MD is inversely associated with adiposity [58], IR [59], and risk of type 2 diabetes mellitus [60] and cardiovascular disease [61]. On this basis, it was conceivable that MD might be considered to be one of the best nutritional strategies also for the management of PCOS women.

The PREDIMED score results from a brief and useful 14-item questionnaire, validated for the assessment of MD adherence [34,62]. The PREDIMED questionnaire is a versatile and practical tool for obtaining a general and complete feedback about the participant dietary pattern in a short time [62,63]. According to the 14 items of the PREDIMED questionnaire, we reported that the intake of fish, nuts, vegetables, and legumes was lower in PCOS women when compared to the control group. A further novel finding of our study is the inverse association between the degree of adherence to the MD, assessed by PREDIMED score and the clinical severity of PCOS. Interestingly, this relationship was still significant when the data were adjusted for BMI and total energy intake, thus suggesting that MD might play an independent role in mitigating the PCOS phenotype, probably through its anti-inflammatory potential. In addition, we observed that, among the MD foods, the PCOS women consumed less extra-virgin olive oil and nuts than the controls. These results seem to be of particular interest when considering the nutraceutical potential of several MD foods, including extra-virgin olive oil, which is one of the main components of this dietary pattern. In extra-virgin olive oil, indeed, a large body of phenolic compounds has been identified and, among these, oleocanthal has been recognized as a potent anti-inflammatory agent [64], due to its analogy with the chemical structure of ibuprofen [16]. On this basis, it is tempting to speculate that the long-term consumption of extra-virgin olive oil might contribute to slow the progression of the inflammatory status, thus improving both insulin sensitivity and compensatory hyperinsulinemia also in PCOS [65].

However, as diet is complex miscellanea of different foods and nutrients, the nutritional approach should be based on a meticulous dietary assessment, in terms of quantity and quality, like the one that was provided by the seven-day food records, the gold standard in self-administered food frequency questionnaires. The analysis of seven-day food records showed that PCOS women consumed a higher amount of high glycemic index carbohydrates, lower fiber, MUFA, and n-3 PUFA as compared to the control group. These results are in line with previous studies reporting that a high intake of simple carbohydrates exerts a pro-inflammatory effect, as demonstrated by increased CRP levels [66], and they are responsible for increased oxidative stress, caused by postprandial hyperglycemia [67]. This pro-inflammatory status might result in exacerbating IR, hyperandrogenism and inflammation in women with PCOS [65]. Interestingly, it has been reported that specific nutrients, such as glucose, are able to stimulate the production of androgen by ovarian and directly promote inflammation in PCOS women [65,68]. 

In parallel to carbohydrates, dietary fat, and in particular MUFA and n-3 PUFA, are also implicated in the PCOS pathogenesis. In our study, we found that the lowest intake of MUFA was among the three main predictors of the highest testosterone levels. The association between low MUFA consumption in PCOS women is in agreement with the clinical trial performed by Kalgaonkar et al. [69]. MUFA are considered as a healthy dietary fat and, traditionally, the beneficial effects of extra-virgin olive oil have been attributed to its high MUFA content (oleic acid) as it protects lipoproteins and cellular membranes from oxidative damage [70]. Most of the studies investigating the effects of n-3 PUFA in PCOS women are clinical trials in which n-3 PUFA are administered as supplementation. A recent meta-analysis of nine studies involving 591 subjects demonstrated that n-3 PUFA supplementation improves the IR, reduces total cholesterol and triglycerides serum levels, and increases adiponectin levels, which suggests that n-3 PUFA supplementation should be recommended for the metabolic management of PCOS women [71]. Among these effects, however, the anti-inflammatory activity of n-3 PUFA is undoubtedly one of the most important concern for the management of PCOS women. Jamilian et al. [72] demonstrated that 12-week treatment with 1 g n-3 PUFA daily up-regulated PPAR-γ expression and down-regulated IL-8 and TNF-α expression in peripheral blood mononuclear cells.

It is clear that unhealthy diets, besides the effects on inflammation and IR, could negatively affect the body composition, which, in turn, is also responsible for worsening of the PCOS clinical severity. Besides BMI, limited data are available on the body composition of PCOS women. No difference in terms of body composition assessed by BIA has been detected in a pilot study that was performed on adolescents with and without diagnosis of PCOS [11]. On the contrary, Ezeh et al. found that, in adult PCOS women, the alteration in the fat-to-lean mass ratio that was evaluated by BIA could represent a possible mechanism determining the increased IR in females [22]. Data herein presented show that PCOS women have lower PhA and FFM as compared to age- and BMI-matched controls. These data appear of interest considering the well-established relationship between higher adiposity, impaired insulin sensitivity, and inflammatory status, which are all factors that are tightly involved in the pathogenesis of PCOS. BIA is a common and validated method for the evaluation of body composition in the clinical practice, with a high agreement with Dual X-ray Absorptiometry, also among patients with severe obesity [21]. Although changes in the hydration status might cause errors in BIA measures, PhA appears to represent a more suitable measurement, as it derives from the relationship between resistance and Xc, two direct BIA measures [52] and its validity is also proven in cases of changes in hydration status, such as in obesity and inflammation [73,74,75]. The PhA is an indicator of the general health of cells [76] and water distribution [77]. Interestingly, PhA is related to body mass [78] and ECW/ICW ratio [79], but it also represents as a valid parameter of the inflammation status in cutaneous inflammatory diseases [46]. In our study, PhA was negatively associated not only with the inflammatory status, but also with the hyperandrogenemia. Although these correlations were not kept after adjustment for BMI and total energy intake, our results suggested that PhA might be considered to be a marker of the severity of the PCOS.

We are aware that there are some limitations in the current study. First, the cross-sectional nature of this study did not allow for any statements on a causal relationship between MD and clinical severity of PCOS, especially when considering the adherence and dietary patterns through a previous lifespan. Second, the sample size is relatively small and dietary intake is related to a larger number of other lifestyle factors. Nevertheless, the sample size was calculated using 95% power; in addition, the association between MD and testosterone levels was independent of the effects of other health-related behaviours (BMI and total energy intake). Third, the PREDIMED score, although being easy to perform by the participants, only allows relative but no absolute statements regarding the degree of adherence to the MD. The PREDIMED questionnaire was face-to-face administered and not self-reported in order to minimize any bias related to the filling of the questionnaire. In addition, to avoid the inter-operator variability, only one expert Nutritionist evaluated the anthropometric measures, administered the PREDIMED questionnaire and seven-day food records, and assesses, executes, and interprets BIA measurements. Anyway, the suggested cut-point for the PREDIMED score for identifying the highest of testosterone levels should be viewed with caution until results of studies in larger population samples have become available to perform an appropriate cross-validation.

Nevertheless, the main strength of this study is the use of the seven-day food records. This tool, which is considered the “gold standard” in validation studies of different types of self-administered food frequency questionnaires, allows for more accurate measurement of the real dietary and macronutrient intakes as compared to those that were obtained by retrospective food frequency questionnaires [80]. Moreover, we increased the homogeneity of the patient sample by including only treatment-naïve PCOS women, and matched controls in order to improve the power of the study.

## 5. Conclusions

In summary, in the present study, we reported for the first time in women with PCOS: (i) a direct association between the adherence to MD and PCOS; (ii) a different body composition that is characterized by low PhA and fat-free mass. These data could support a therapeutic role of single foods and nutrients of the Mediterranean dietary pattern in the PCOS, by contributing to reduce the inflammatory status that paves the way for IR and hyperandrogenemia. In addition, the role for PhA as a useful marker of the clinical severity of PCOS is strongly suggested. Our data propose that the strategic relevance of the nutritional assessment and body composition evaluation of women with PCOS should be considered as the first and most important step in the management of this syndrome.

## Figures and Tables

**Table 1:** Lifestyle habits, anthropometric measurements, adherence to the Mediterranean diet (MD), inflammatory parameter, hormonal, and biochemical parameters and clinical hyperandrogenism of the case-patients and the subjects matched for age and BMI, serving as control group.

Parameters	PCOS Patients *n* = 112	Control Group *n* = 112	*p* Values
Lifestyle Habits			
Age (years)	24.21 ± 5.47	24.07 ± 5.05	0.721
Smoking			χ^2^ = 0.10, *p* = 0.756
Yes (*n*, %)	29, 25.9%	26, 23.2%	
No (*n*, %)	83, 74.1%	86, 76.8%	
Physical activity			χ^2^ = 0.08, *p* = 0.777
Sedentary (*n*, %)	76, 67.9%	73, 65.2%	
Moderate (*n*, %)	36, 32.1%	39, 34.8%	
Anthropometric measurements			
BMI (kg/m^2^)	30.95 ± 5.66	30.76 ± 5.60	0.273
Normal-weight (*n*, %)	24, 21.4%	24, 21.4%	**χ^2^ = 0.00, *p* = 1.000**
Over-weight (*n*, %)	29, 25.9%	29, 25.9%	
Obesity I (*n*, %)	24, 21.4%	24, 21.4%	
Obesity II (*n*, %)	35, 31.3%	35, 31.3%	
WC (cm)	101.09 ± 16.29	92.54 ± 14.17	**<0.001**
WC< cut-off	20, 17.9%	43, 38.4%	χ^2^ = 10.69, *p* = 0.001
WC > cut-off	92, 82.1%	69, 61.6%	
Adherence to the MD			
PREDIMED score	6.97 ± 2.72	8.12 ± 2.80	**<0.001**
Low adherence to the MD	37, 33.0%	21, 18.8%	χ^2^ = 5.24, ***p* = 0.022**
Average adherence to the MD	53, 47.3%	58, 51.8%	χ^2^ = 0.29, *p* = 0.593
High adherence to the MD	22, 19.6%	33, 29.5%	χ^2^ = 2.41, *p* = 0.121
Inflammatory parameter			
CRP levels (ng/mL)	1.03 ± 0.84	0.58 ± 0.44	**<0.001**
Hormonal and biochemical parameters			
Testosterone (ng/dL)	28.51 ± 9.82	10.21 ± 4.39	**<0.001**
Insulin (μU/mL)	11.61 ± 12.97	6.19 ± 7.79	**<0.001**
Fasting glucose (mg/dL)	94.92 ± 11.70	89.03 ± 10.89	**<0.001**
HoMA-IR	2.90 ± 3.59	1.45 ± 1.95	**<0.001**
Clinical Hyperandrogenism			
Ferriman-Gallwey score	15.64 ± 9.69	2.47 ± 1.68	**<0.001**

The PCOS patients exhibited statistically significant differences compared to controls for WC, PREDIMED score, CRP levels, hormonal, biochemical parameters and clinical hyperandrogenism. Results are expressed as mean ± SD. The chi-square (χ^2^) test was used to determine the significance of differences in smoking habit, physical activity, BMI categories, cut-off of WC and PREDIMED categories between the two groups. A *p* value in bold type denotes a significant difference (*p* < 0.05). PCOS, Polycystic Ovarian Syndrome; BMI, Body Mass Index; WC, Waist Circumference; MD, Mediterranean Diet, PREDIMED, PREvención con DIetaMEDiterránea; CRP, C-reactive Protein; HoMA-IR, Homeostasis model assessment insulin resistance.

**Table 2:** Response frequency of dietary components included in the PREvención con DIetaMEDiterránea (PREDIMED) questionnaire of the polycystic ovary syndrome (PCOS) patients and control group.

Questions PREDIMED Questionnaire	PCOS Patients *n* = 112		Control Group *n* = 112		χ^2^	*p* Values
	*n*	%	*n*	%		
Use of extra-virgin olive oil as main culinary lipid	88	78.6	108	96.4	14.74	**<0.001**
Extra virgin olive oil >4 tablespoons	64	57.1	55	49.1	1.15	0.284
Vegetables ≥2 servings/day	54	48.2	62	55.4	0.87	0.349
Fruits ≥3 servings/day	67	59.8	66	58.9	0.01	1.000
Red/processed meats <1/day	62	55.4	57	50.9	0.28	0.592
Butter, cream, margarine <1/day	47	42.0	55	49.1	0.88	0.348
Soda drinks <1/day	53	47.3	58	51.8	0.28	0.593
Wine glasses ≥ 7/week	30	26.8	35	31.3	0.35	0.555
Legumes ≥ 3/week	60	53.6	81	72.3	7.66	**0.006**
Fish/seafood ≥ 3/week	35	31.3	76	67.9	28.57	**<0.001**
Commercial sweets and confectionery ≤ 2/week	59	52.7	56	50.0	0.07	0.789
Tree nuts ≥ 3/week	31	27.7	76	67.6	34.64	**<0.001**
Poultry more than red meats	63	56.3	51	45.5	2.16	0.142
Use of sofrito sauce ≥ 2/week	68	60.7	73	65.2	0.31	0.580

PCOS patients exhibited statistically significant differences in use of extra-virgin olive oil as main culinary lipid, legumes, fish/seafood, and nuts consumption, compared with control group. Results are expressed as number and percentage of responses obtained with PREDIMED questionnaire. The differences were analysed by χ^2^ test. A *p* value in bold type denotes a significant difference (*p* < 0.05). PREDIMED, PREvención con DIetaMEDiterránea; PCOS, Polycystic Ovarian Syndrome.

**Table 3:** Daily energy, nutrients intake and bioelectrical impedance analysis (BIA) parameters of the PCOS patients and control group.

Parameters	PCOS Patients *n* = 112	Control Group *n* = 112	*p*-Value
Dietary Intake			
Total energy (kcal)	2245.31 ± 290.75	2254.84 ± 272.37	0.711
Protein (g of total kcal)	86.98 ± 10.15	88.43 ± 9.96	0.261
Carbohydrate (g of total kcal)	307.98 ± 42.03	310.47 ± 37.42	0.518
Complex (g of total kcal)	174.21 ± 25.43	191.48 ± 23.60	**<0.001**
Simple (g of total kcal)	133.77 ± 34.01	118.99 ± 33.62	**<0.001**
Fiber (g/day)	15.43 ± 3.66	17.22 ± 4.19	**<0.001**
Fat (g of total kcal)	73.94 ± 13.59	70.07 ± 10.73	**<0.001**
SFA (g of total kcal)	24.55 ± 7.51	17.39 ± 10.71	**<0.001**
Unsaturated fat (g of total kcal)	49.38 ± 8.63	52.67 ± 8.12	**0.002**
MUFA (g of total kcal)	38.21 ± 4.56	43.68 ± 5.86	**<0.001**
PUFA (g of total kcal)	11.16 ± 6.88	8.99 ± 4.69	**0.005**
n-6 PUFA (g/day)	7.82 ± 6.83	4.67 ± 3.87	**<0.001**
n-3 PUFA (g/day)	3.34 ± 2.24	4.32 ± 3.29	**<0.001**
Body Composition			
R (Ω)	488.71 ± 82.59	477.73 ± 71.79	0.289
Xc (Ω)	49.05 ± 10.09	51.58 ± 9.58	**0.028**
PhA (°)	5.76 ± 0.71	6.20 ± 0.79	**<0.001**
FM (%)	34.47 ± 9.63	29.75 ± 9.88	**<0.001**
FFM (%)	65.44 ± 9.67	69.89 ± 10.07	**<0.001**
BCM (%)	49.92 ± 8.67	52.03 ± 9.92	0.082
TBW (%)	47.97 ± 7.06	49.99 ± 7.44	**0.001**
ECW (%)	47.20 ± 3.50	45.85 ± 3.92	**0.004**
ICW (%)	52.81 ± 3.51	54.15 ± 3.92	**0.004**

PCOS patients exhibited statistically significant differences compared with control group for dietary intake and BIA parameters. In spite of no differences in energy intake between the two groups, PCOS patients have the lowest intake of complex carbohydrate, fiber, unsaturated fatty acids, MUFA and n-3 PUFA, and higher quantity of simple carbohydrate, total fat, SFA, PUFA and n-6 PUFA than control group. In addition, considering BIA parameters, PCOS patients had a lowest values of Xc, PhA, FFM, TBW and ICW, and the highest values of FM and ECW. Results are expressed as mean ± SD. Differences between groups were analysed by paired Student’s *t* test. A *p* value in bold type denotes a significant difference (*p* < 0.05). BIA, Bioelectrical Impedance Analysis, PCOS, Polycystic Ovarian Syndrome; SFA, Saturated Fatty Acids; MUFA, MonoUnsaturated Fatty Acids; PUFA, PolyUnsaturated Fatty Acids; R, Resistance; Xc, Reactance; PhA, Phase angle; FM, Fat Mass, FFM, Fat-Free Mass, BCM, Body Mass Cell, TBW, Total Body Water, ECW, Extra-cellular Water, ICW, Intra-cellular Water.

**Table 4:** Age, anthropometric measurements, adherence to the MD, inflammatory parameter, dietary intake and body composition of the case-patients above and below the median of testosterone.

Parameters	Testosterone (ng/dL)		
	< 22.27 ng/dL	> 22.27 ng/dL	*p*-Value
	*n* = 56	*n* = 56	
Age (years)	24.02 ± 5.74	24.41 ± 5.21	0.706
BMI (kg/m^2^)	27.39 ± 4.88	34.53 ± 3.85	**<0.001**
Normal-weight (*n*, %)	24, 42.9%	0, 0%	**<0.001**
Over-weight (*n*, %)	20, 35.7%	9, 16.1%	**0.031**
Obesity I (*n*, %)	3, 5.4%	21, 37.5%	**<0.001**
Obesity II (*n*, %)	9, 16.1%	26, 46.4%	**0.001**
WC (cm)	92.76 ± 12.69	109.42 ± 15.29	**<0.001**
WC > cut-off	37, 66.1%	55, 98.2%	**<0.001**
Adherence to the MD			
PREDIMED score	8.61 ± 2.37	5.34 ± 1.96	**<0.001**
Low adherence to the MD	7, 12.5%	0, 0%	**<0.001**
Average adherence to the MD	27, 48.2%	30, 53.6%	0.999
High adherence to the MD	22, 39.3%	26, 46.4%	**<0.001**
Inflammatory parameter			
CRP levels (ng/dL)	0.51 ± 0.31	1.56 ± 0.88	**<0.001**
Hormonal and biochemical parameters			
Insulin (μU/mL)	6.06 ± 7.85	17.16 ± 14.67	**<0.001**
Fasting glucose (mg/dL)	90.36 ± 10.97	99.48 ± 10.64	**<0.001**
HoMA-IR	1.39 ± 2.11	4.41 ± 4.13	**<0.001**
Dietary Intake			
Total energy (kcal)	2141.83 ± 230.24	2348.78 ± 309.52	**<0.001**
Protein (g of total kcal)	88.01 ± 10.69	85.96 ± 9.55	0.287
Carbohydrate (g of total kcal)	292.29 ± 31.87	323.67 ± 45.26	**<0.001**
Complex (g of total kcal)	173.01 ± 20.24	175.41 ± 29.88	0.619
Simple (g of total kcal)	119.28 ± 26.68	148.25 ± 34.58	**<0.001**
Fiber (g/day)	17.08 ± 3.48	13.79 ± 3.04	**<0.001**
Fat (g of total kcal)	68.96 ± 10.70	78.92 ± 14.42	**<0.001**
SFA (g of total kcal)	20.51 ± 6.14	28.60 ± 6.54	**<0.001**
Unsaturated fat (g of total kcal)	48.45 ± 6.73	50.32 ± 10.16	0.254
MUFA (g of total kcal)	39.49 ± 3.45	36.94 ± 5.18	**0.003**
PUFA (g of total kcal)	8.96 ± 5.10	13.38 ± 7.71	**0.001**
n-6 PUFA (g/day)	5.51 ± 4.92	10.14 ± 7.69	**<0.001**
n-3 PUFA (g/day)	3.44 ± 2.28	3.23 ± 2.21	0.631
Body Composition			
R (Ω)	504.93 ± 73.69	472.50 ± 88.31	**0.037**
Xc (Ω)	52.27 ± 8.96	45.83 ± 10.20	**0.001**
PhA (°)	5.94 ± 0.65	5.57 ± 0.72	**0.006**
FM (%)	30.07 ± 9.19	38.86 ± 7.99	**<0.001**
FFM (%)	69.74 ± 9.33	61.14 ± 7.99	**<0.001**
BCM (%)	51.53 ± 7.95	48.31 ± 9.12	**0.049**
TBW (%)	51.19 ± 6.72	44.76 ± 5.85	**<0.001**
ECW (%)	46.29 ± 2.99	48.13 ± 3.76	**0.005**
ICW (%)	53.74 ± 3.02	51.88 ± 3.74	**<0.001**

The PCOS patients were examined by stratifying above and below the median of testosterone. PCOS patients below the median of testosterone had the worse anthropometric measurements, adherence to the MD and CRP levels, dietary pattern and body composition compared to the control group. Results are expressed as mean ± SD. Differences between groups were analysed by paired Student’s *t* test. A *p* value in bold type denotes a significant difference (*p* < 0.05). MD, Mediterranean Diet; BMI, Body Mass Index; WC, Waist Circumference; PREDIMED, PREvención con DIetaMEDiterránea; CRP, C-reactive Protein; HoMA-IR, Homeostasis model assessment insulin resistance; SFA, Saturated Fatty Acids; MUFA, MonoUnsaturated Fatty Acids; PUFA, PolyUnsaturated Fatty Acids; R, Resistance; Xc, Reactance; PhA, Phase angle; FM, Fat Mass, FFM, Fat-free Mass, BCM, Body Mass Cell, TBW, Total Body Water, ECW, Extra-cellular Water, ICW, Intra-cellular Water.

**Table 5:** Age, anthropometric measurements, adherence to the MD, inflammatory parameter, dietary intake and body composition of the case-patients and the control group.

	Testosterone Levels (ng/dL)			
Parameters	Simple Correlation		After Adjusted for BMI and Total Energy	
	r	*p*-Value	r	*p*-Value
Age (years)	−0.034	0.725	−0.057	0.555
BMI (kg/m^2^)	0.720	**<0.001**	-	-
WC (cm)	0.722	**<0.001**	0.399	**<0.001**
PREDIMED score	−0.716	**<0.001**	−0.357	**<0.001**
CRP levels (ng/dL)	0.774	**<0.001**	0.677	**<0.001**
Insulin (μU/mL)	0.721	**<0.001**	0.490	**<0.001**
Fasting glucose (mg/dL)	0.517	**<0.001**	0.176	0.066
HoMA-IR	0.792	**<0.001**	0.485	**<0.001**
Total energy (kcal)	0.492	**<0.001**		
Protein (g of total kcal)	−0.167	0.078	−0.264	**0.005**
Carbohydrate (g of total kcal)	0.490	**<0.001**	−0.003	0.979
Complex (g of total kcal)	−0.0.18	0.849	−0.365	**<0.001**
Simple (g of total kcal)	0.620	**<0.001**	0.419	**<0.001**
Fiber (g/day)	−0.575	**<0.001**	−0.381	**<0.001**
Fat (g of total kcal)	0.551	**<0.001**	0.158	0.100
SFA (g of total kcal)	0.691	**<0.001**	0.321	**0.001**
Unsaturated fat (g of total kcal)	0.267	**0.004**	−0.018	0.850
MUFA (g of total kcal)	−0.244	**0.010**	−0.444	**<0.001**
PUFA (g of total kcal)	0.497	**<0.001**	0.288	**0.002**
n-6 PUFA (g/day)	0.522	**<0.001**	0.412	**<0.001**
n-3 PUFA (g/day)	−0.068	0.476	−0.314	**0.001**
R (Ω)	−0.253	**0.007**	−0.071	0.460
Xc (Ω)	−0.482	**<0.001**	−0.152	0.114
PhA (°)	−0.452	**<0.001**	−0.192	0.095
FM (%)	0.483	**<0.001**	−0.121	0.207
FFM (%)	−0.474	**<0.001**	0.117	0.223
BCM (%)	−0.322	**0.001**	0.005	0.957
TBW (%)	−0.483	**<0.001**	0.121	0.209
ECW (%)	0.474	**0.001**	0.179	0.061
ICW (%)	−0.475	**0.001**	−0.181	0.058

Correlations between testosterone levels with anthropometric parameters, adherence to the MD, CRP levels, dietary intake and body composition of the PCOS women. Also after adjusting for BMI, and total energy intake, testosterone levels showed significant correlations with PREDIMED score and some dietary nutrients. A *p* value in bold type denotes a significant difference (*p* < 0.05). BMI, Body Mass Index; WC, Waist Circumference; PREDIMED, PREvención con DIetaMEDiterránea; CRP, C-reactive Protein; HoMA-IR, Homeostasis model assessment insulin resistance; SFA, Saturated Fatty Acids; MUFA, MonoUnsaturated Fatty Acids; PUFA, PolyUnsaturated Fatty Acids; R, Resistance; Xc, Reactance; PhA, Phase angle; FM, Fat Mass, FFM, Fat-free Mass, BCM, Body Mass Cell, TBW, Total Body Water, ECW, Extra-cellular Water, ICW, Intra-cellular Water.

**Table 6:** Multiple regression analysis model (stepwise method) with the Testosterone as dependent variable to estimate the predictive value of food items of WC, PREDIMED score, CRP levels, HoMA-IR and nutrients intake (protein, complex carbohydrate, simple carbohydrate, SFA, MUFA, PUFA, n-6 PUFA, n-3 PUFA, fiber).

Parameters	Multiple Regression Analysis			
Model 1	R^2^	β	t	*p* Value
**CRP levels**	0.704	−0.841	16.28	**<0.001**
**PREDIMED SCORE**	0.724	−0.212	−2.96	**<0.001**
**MUFA (g of total kcal)**	0.738	−0.133	−2.61	**0.001**
Variable excluded: WC, HoMA-IR, protein, complex carbohydrate, simple carbohydrate, SFA, PUFA, n-6 PUFA, n-3 PUFA, fiber.				

Between adherence to the MD and dietary nutrients intake, testosterone levels were well predicted by PREDIMED score and MUFA intake. A *p* value in bold type denotes a significant difference (*p* < 0.05). CRP, C-reactive Protein; PREDIMED, PREvención con DIetaMEDiterránea; MUFA, MonoUnsaturated Fatty Acids; WC, Waist Circumference; HoMA-IR, Homeostasis model assessment insulin resistance; SFA, Saturated Fatty Acids; PolyUnsaturated Fatty Acids.

## Abbreviations

PCOS, Polycystic ovary syndrome; IR, insulin resistance; MD, Mediterranean diet; PUFA, polyunsaturated fat acid; BMI, body mass index; BIA, bioelectrical impedance analysis; FM, fat mass; PhA, phase angle; SD, standard deviation; PREDIMED, PREvención con DIetaMEDiterránea; WC, waist circumference; R, Resistance; Xc, Reactance; CV, coefficient of variation; HoMA, Homeostasis model assessment; CRP, C-reactive protein; SFA, saturated fat acid; MUFA, monounsaturated fat acid; ROC, receiver operator characteristic; AUC, area under the curve; IC, confidence interval; FFM, fat-free mass; TBW, total body water; ICW, intracellular water; ECW, extracellular water; BCM, Body Mass Cell.

## References

[1] Laven J.S., Imani B., Eijkemans M.J., Fauser B.C. (2002). New approach to polycystic ovary syndrome and other forms of anovulatory infertility. Obstet. Gynecol. Surv..

[2] March W.A., Moore V.M., Willson K.J., Phillips D.I., Norman R.J., Davies M.J. (2010). The prevalence of polycystic ovary syndrome in a community sample assessed under contrasting diagnostic criteria. Hum. Reprod..

[3] Rotterdam E.A., ASRM-Sponsored PCOS Consensus Workshop Group (2004). Revised 2003 consensus on diagnostic criteria and long-term health risks related to polycystic ovary syndrome (PCOS). Hum. Reprod..

[4] Amiri M., Ramezani Tehrani F., Nahidi F., Bidhendi Yarandi R., Behboudi-Gandevani S., Azizi F. (2017). Association between biochemical hyperandrogenism parameters and Ferriman-Gallwey score in patients with polycystic ovary syndrome: A systematic review and meta-regression analysis. Clin. Endocrinol. (Oxf.).

[5] Diamanti-Kandarakis E., Economou F., Palimeri S., Christakou C. (2010). Metformin in polycystic ovary syndrome. Ann. N. Y. Acad. Sci..

[6] Gonzalez F. (2012). Inflammation in Polycystic Ovary Syndrome: Underpinning of insulin resistance and ovarian dysfunction. Steroids.

[7] Deswal R., Yadav A., Dang A.S. (2018). Sex hormone binding globulin—An important biomarker for predicting PCOS risk: A systematic review and meta-analysis. Syst. Biol. Reprod. Med..

[8] Moghetti P., Tosi F., Castello R., Magnani C.M., Negri C., Brun E., Furlani L., Caputo M., Muggeo M. (1996). The insulin resistance in women with hyperandrogenism is partially reversed by antiandrogen treatment: Evidence that androgens impair insulin action in women. J. Clin. Endocrinol. Metab..

[9] Lord J., Thomas R., Fox B., Acharya U., Wilkin T. (2006). The central issue? Visceral fat mass is a good marker of insulin resistance and metabolic disturbance in women with polycystic ovary syndrome. BJOG Int. J. Obstet. Gynaecol..

[10] Fauser B.C., Tarlatzis B.C., Rebar R.W., Legro R.S., Balen A.H., Lobo R., Carmina E., Chang J., Yildiz B.O., Laven J.S. (2012). Consensus on women’s health aspects of polycystic ovary syndrome (PCOS): The Amsterdam ESHRE/ASRM-Sponsored 3rd PCOS Consensus Workshop Group. Fertil. Steril..

[11] Geronikolou S.A., Bacopoulou F., Cokkinos D. (2017). Bioimpedance Measurements in Adolescents with Polycystic Ovary Syndrome: A Pilot Study. Adv. Exp. Med. Biol..

[12] Teede H.J., Misso M.L., Costello M.F., Dokras A., Laven J., Moran L., Piltonen T., Norman R.J., International P.N. (2018). Recommendations from the international evidence-based guideline for the assessment and management of polycystic ovary syndrome. Hum. Reprod..

[13] Willett W.C., Sacks F., Trichopoulou A., Drescher G., Ferro-Luzzi A., Helsing E., Trichopoulos D. (1995). Mediterranean diet pyramid: A cultural model for healthy eating. Am. J. Clin. Nutr..

[14] Desai M.S., Seekatz A.M., Koropatkin N.M., Kamada N., Hickey C.A., Wolter M., Pudlo N.A., Kitamoto S., Terrapon N., Muller A. (2016). A Dietary Fiber-Deprived Gut Microbiota Degrades the Colonic Mucus Barrier and Enhances Pathogen Susceptibility. Cell.

[15] Athar M., Back J.H., Kopelovich L., Bickers D.R., Kim A.L. (2009). Multiple molecular targets of resveratrol: Anti-carcinogenic mechanisms. Arch. Biochem. Biophys..

[16] Cicerale S., Breslin P.A., Beauchamp G.K., Keast R.S. (2009). Sensory characterization of the irritant properties of oleocanthal, a natural anti-inflammatory agent in extra virgin olive oils. Chem. Senses.

[17] Singh U.P., Singh N.P., Singh B., Hofseth L.J., Taub D.D., Price R.L., Nagarkatti M., Nagarkatti P.S. (2012). Role of resveratrol-induced CD11b(+) Gr-1(+) myeloid derived suppressor cells (MDSCs) in the reduction of CXCR3(+) T cells and amelioration of chronic colitis in IL-10(-/-) mice. Brain Behav. Immun..

[18] Rajaram S., Connell K.M., Sabate J. (2010). Effect of almond-enriched high-monounsaturated fat diet on selected markers of inflammation: A randomised, controlled, crossover study. Br. J. Nutr..

[19] Morken T., Bohov P., Skorve J., Ulvik R., Aukrust P., Berge R.K., Livden J.K. (2011). Anti-inflammatory and hypolipidemic effects of the modified fatty acid tetradecylthioacetic acid in psoriasis—A pilot study. Scand. J. Clin. Lab. Investig..

[20] Douglas C.C., Norris L.E., Oster R.A., Darnell B.E., Azziz R., Gower B.A. (2006). Difference in dietary intake between women with polycystic ovary syndrome and healthy controls. Fertil. Steril..

[21] Savastano S., Belfiore A., Di Somma C., Mauriello C., Rossi A., Pizza G., De Rosa A., Prestieri G., Angrisani L., Colao A. (2010). Validity of bioelectrical impedance analysis to estimate body composition changes after bariatric surgery in premenopausal morbidly women. Obes. Surg..

[22] Ezeh U., Pall M., Mathur R., Azziz R. (2014). Association of fat to lean mass ratio with metabolic dysfunction in women with polycystic ovary syndrome. Hum. Reprod..

[23] Hestiantoro A., Kapnosa Hasani R.D., Shadrina A., Situmorang H., Ilma N., Muharam R., Sumapraja K., Wiweko B. (2018). Body fat percentage is a better marker than body mass index for determining inflammation status in polycystic ovary syndrome. Int. J. Reprod. Biomed. (Yazd).

[24] Legro R.S., Arslanian S.A., Ehrmann D.A., Hoeger K.M., Murad M.H., Pasquali R., Welt C.K., Endocrine S. (2013). Diagnosis and treatment of polycystic ovary syndrome: An Endocrine Society clinical practice guideline. J. Clin. Endocrinol. Metab..

[25] Romualdi D., Versace V., Tagliaferri V., De Cicco S., Immediata V., Apa R., Guido M., Lanzone A. (2019). The resting metabolic rate in women with polycystic ovary syndrome and its relation to the hormonal milieu, insulin metabolism, and body fat distribution: A cohort study. J. Endocrinol. Investig..

[26] Barrea L., Annunziata G., Muscogiuri G., Di Somma C., Laudisio D., Maisto M., de Alteriis G., Tenore G.C., Colao A., Savastano S. (2018). Trimethylamine-N-oxide (TMAO) as Novel Potential Biomarker of Early Predictors of Metabolic Syndrome. Nutrients.

[27] Barrea L., Annunziata G., Muscogiuri G., Laudisio D., Di Somma C., Maisto M., Tenore G.C., Colao A., Savastano S. (2019). Trimethylamine N-oxide, Mediterranean diet, and nutrition in healthy, normal-weight adults: Also a matter of sex?. Nutrition.

[28] Muscogiuri G., Barrea L., Di Somma C., Altieri B., Vecchiarini M., Orio F., Spinosa T., Colao A., Savastano S. (2019). Patient empowerment and the Mediterranean diet as a possible tool to tackle prediabetes associated with overweight or obesity: A pilot study. Hormones (Athens).

[29] Savastano S., Di Somma C., Colao A., Barrea L., Orio F., Finelli C., Pasanisi F., Contaldo F., Tarantino G. (2015). Preliminary data on the relationship between circulating levels of Sirtuin 4, anthropometric and metabolic parameters in obese subjects according to growth hormone/insulin-like growth factor-1 status. Growth Horm. IGF Res. Off. J. Growth Horm. Res. Soc. Int. IGF Res. Soc..

[30] Savanelli M.C., Scarano E., Muscogiuri G., Barrea L., Vuolo L., Rubino M., Savastano S., Colao A., Di Somma C. (2016). Cardiovascular risk in adult hypopituitaric patients with growth hormone deficiency: Is there a role for vitamin D?. Endocrine.

[31] Barrea L., Muscogiuri G., Annunziata G., Laudisio D., de Alteriis G., Tenore G.C., Colao A., Savastano S. (2019). A New Light on Vitamin D in Obesity: A Novel Association with Trimethylamine-N-Oxide (TMAO). Nutrients.

[32] Body Mass Index. http://www.euro.who.int/en/health-topics/disease-prevention/nutrition/a-healthy-lifestyle/body-mass-index-bmi.

[33] Anthropometry Procedures Manual. http://www.cdc.gov/nchs/data/nhanes/nhanes_11_12/Anthropometry_Procedures_Manual.pdf.

[34] Martinez-Gonzalez M.A., Garcia-Arellano A., Toledo E., Salas-Salvado J., Buil-Cosiales P., Corella D., Covas M.I., Schroder H., Aros F., Gomez-Gracia E. (2012). A 14-item Mediterranean diet assessment tool and obesity indexes among high-risk subjects: The PREDIMED trial. PLoS ONE.

[35] Barrea L., Balato N., Di Somma C., Macchia P.E., Napolitano M., Savanelli M.C., Esposito K., Colao A., Savastano S. (2015). Nutrition and psoriasis: Is there any association between the severity of the disease and adherence to the Mediterranean diet?. J. Transl. Med..

[36] Barrea L., Tarantino G., Somma C.D., Muscogiuri G., Macchia P.E., Falco A., Colao A., Savastano S. (2017). Adherence to the Mediterranean Diet and Circulating Levels of Sirtuin 4 in Obese Patients: A Novel Association. Oxid. Med. Cell. Longev..

[37] Barrea L., Altieri B., Muscogiuri G., Laudisio D., Annunziata G., Colao A., Faggiano A., Savastano S. (2018). Impact of Nutritional Status on Gastroenteropancreatic Neuroendocrine Tumors (GEP-NET) Aggressiveness. Nutrients.

[38] Barrea L., Muscogiuri G., Di Somma C., Tramontano G., De Luca V., Illario M., Colao A., Savastano S. (2019). Association between Mediterranean diet and hand grip strength in older adult women. Clin. Nutr..

[39] Barrea L., Macchia P.E., Tarantino G., Di Somma C., Pane E., Balato N., Napolitano M., Colao A., Savastano S. (2015). Nutrition: A key environmental dietary factor in clinical severity and cardio-metabolic risk in psoriatic male patients evaluated by 7-day food-frequency questionnaire. J. Transl. Med..

[40] Barrea L., Di Somma C., Macchia P.E., Falco A., Savanelli M.C., Orio F., Colao A., Savastano S. (2017). Influence of nutrition on somatotropic axis: Milk consumption in adult individuals with moderate-severe obesity. Clin. Nutr..

[41] Savanelli M.C., Barrea L., Macchia P.E., Savastano S., Falco A., Renzullo A., Scarano E., Nettore I.C., Colao A., Di Somma C. (2017). Preliminary results demonstrating the impact of Mediterranean diet on bone health. J. Transl. Med..

[42] Barrea L., Muscogiuri G., Di Somma C., Annunziata G., Megna M., Falco A., Balato A., Colao A., Savastano S. (2018). Coffee consumption, metabolic syndrome and clinical severity of psoriasis: Good or bad stuff?. Arch. Toxicol..

[43] Turconi G., Guarcello M., Berzolari F.G., Carolei A., Bazzano R., Roggi C. (2005). An evaluation of a colour food photography atlas as a tool for quantifying food portion size in epidemiological dietary surveys. Eur. J. Clin. Nutr..

[44] (1996). Bioelectrical impedance analysis in body composition measurement: National Institutes of Health Technology Assessment Conference Statement. Am. J. Clin. Nutr..

[45] Savastano S., Barbato A., Di Somma C., Guida B., Pizza G., Barrea L., Avallone S., Schiano di Cola M., Strazzullo P., Colao A. (2012). Beyond waist circumference in an adult male population of Southern Italy: Is there any role for subscapular skinfold thickness in the relationship between insulin-like growth factor-I system and metabolic parameters?. J. Endocrinol. Investig..

[46] Barrea L., Macchia P.E., Di Somma C., Napolitano M., Balato A., Falco A., Savanelli M.C., Balato N., Colao A., Savastano S. (2016). Bioelectrical phase angle and psoriasis: A novel association with psoriasis severity, quality of life and metabolic syndrome. J. Transl. Med..

[47] Barrea L., Muscogiuri G., Macchia P.E., Di Somma C., Falco A., Savanelli M.C., Colao A., Savastano S. (2017). Mediterranean Diet and Phase Angle in a Sample of Adult Population: Results of a Pilot Study. Nutrients.

[48] Barrea L., Fabbrocini G., Annunziata G., Muscogiuri G., Donnarumma M., Marasca C., Colao A., Savastano S. (2018). Role of Nutrition and Adherence to the Mediterranean Diet in the Multidisciplinary Approach of Hidradenitis Suppurativa: Evaluation of Nutritional Status and Its Association with Severity of Disease. Nutrients.

[49] Kyle U.G., Bosaeus I., De Lorenzo A.D., Deurenberg P., Elia M., Manuel Gomez J., Lilienthal Heitmann B., Kent-Smith L., Melchior J.C., Pirlich M. (2004). Bioelectrical impedance analysis-part II: Utilization in clinical practice. Clin. Nutr..

[50] Kushner R.F. (1992). Bioelectrical impedance analysis: A review of principles and applications. J. Am. Coll. Nutr..

[51] Xu Y., Xie X., Duan Y., Wang L., Cheng Z., Cheng J. (2016). A review of impedance measurements of whole cells. Biosens. Bioelectron..

[52] Barbosa-Silva M.C., Barros A.J., Wang J., Heymsfield S.B., Pierson R.N. (2005). Bioelectrical impedance analysis: Population reference values for phase angle by age and sex. Am. J. Clin. Nutr..

[53] Bosy-Westphal A., Danielzik S., Dorhofer R.P., Later W., Wiese S., Muller M.J. (2006). Phase angle from bioelectrical impedance analysis: Population reference values by age, sex, and body mass index. J. Parenter. Enter. Nutr..

[54] Barrea L., Muscogiuri G., Laudisio D., Somma C.D., Salzano C., Pugliese G., Alteriis G., Colao A., Savastano S. (2019). Phase Angle: A Possible Biomarker to Quantify Inflammation in Subjects with Obesity and 25(OH)D Deficiency. Nutrients.

[55] Matthews D.R., Hosker J.P., Rudenski A.S., Naylor B.A., Treacher D.F., Turner R.C. (1985). Homeostasis model assessment: Insulin resistance and beta-cell function from fasting plasma glucose and insulin concentrations in man. Diabetologia.

[56] Ferriman D., Gallwey J.D. (1961). Clinical assessment of body hair growth in women. J. Clin. Endocrinol. Metab..

[57] Brodell L.A., Mercurio M.G. (2010). Hirsutism: Diagnosis and management. Gend. Med..

[58] Boghossian N.S., Yeung E.H., Mumford S.L., Zhang C., Gaskins A.J., Wactawski-Wende J., Schisterman E.F., BioCycle Study G. (2013). Adherence to the Mediterranean diet and body fat distribution in reproductive aged women. Eur. J. Clin. Nutr..

[59] Abiemo E.E., Alonso A., Nettleton J.A., Steffen L.M., Bertoni A.G., Jain A., Lutsey P.L. (2013). Relationships of the Mediterranean dietary pattern with insulin resistance and diabetes incidence in the Multi-Ethnic Study of Atherosclerosis (MESA). Br. J. Nutr..

[60] Koloverou E., Esposito K., Giugliano D., Panagiotakos D. (2014). The effect of Mediterranean diet on the development of type 2 diabetes mellitus: A meta-analysis of 10 prospective studies and 136,846 participants. Metabolism.

[61] Estruch R., Ros E., Salas-Salvado J., Covas M.I., Corella D., Aros F., Gomez-Gracia E., Ruiz-Gutierrez V., Fiol M., Lapetra J. (2013). Primary prevention of cardiovascular disease with a Mediterranean diet. N. Engl. J. Med..

[62] Schroder H., Fito M., Estruch R., Martinez-Gonzalez M.A., Corella D., Salas-Salvado J., Lamuela-Raventos R., Ros E., Salaverria I., Fiol M. (2011). A short screener is valid for assessing Mediterranean diet adherence among older Spanish men and women. J. Nutr..

[63] Tresserra-Rimbau A., Rimm E.B., Medina-Remon A., Martinez-Gonzalez M.A., Lopez-Sabater M.C., Covas M.I., Corella D., Salas-Salvado J., Gomez-Gracia E., Lapetra J. (2014). Polyphenol intake and mortality risk: A re-analysis of the PREDIMED trial. BMC Med..

[64] Parkinson L., Keast R. (2014). Oleocanthal, a phenolic derived from virgin olive oil: A review of the beneficial effects on inflammatory disease. Int. J. Mol. Sci..

[65] Gonzalez F., Sia C.L., Shepard M.K., Rote N.S., Minium J. (2014). The altered mononuclear cell-derived cytokine response to glucose ingestion is not regulated by excess adiposity in polycystic ovary syndrome. J. Clin. Endocrinol. Metab..

[66] Levitan E.B., Cook N.R., Stampfer M.J., Ridker P.M., Rexrode K.M., Buring J.E., Manson J.E., Liu S. (2008). Dietary glycemic index, dietary glycemic load, blood lipids, and C-reactive protein. Metabolism.

[67] Barrea L., Marzullo P., Muscogiuri G., Di Somma C., Scacchi M., Orio F., Aimaretti G., Colao A., Savastano S. (2018). Source and amount of carbohydrate in the diet and inflammation in women with polycystic ovary syndrome. Nutr. Res. Rev..

[68] Gonzalez F. (2015). Nutrient-Induced Inflammation in Polycystic Ovary Syndrome: Role in the Development of Metabolic Aberration and Ovarian Dysfunction. Semin. Reprod. Med..

[69] Kalgaonkar S., Almario R.U., Gurusinghe D., Garamendi E.M., Buchan W., Kim K., Karakas S.E. (2011). Differential effects of walnuts vs almonds on improving metabolic and endocrine parameters in PCOS. Eur. J. Clin. Nutr..

[70] Berbert A.A., Kondo C.R., Almendra C.L., Matsuo T., Dichi I. (2005). Supplementation of fish oil and olive oil in patients with rheumatoid arthritis. Nutrition.

[71] Yang K., Zeng L., Bao T., Ge J. (2018). Effectiveness of Omega-3 fatty acid for polycystic ovary syndrome: A systematic review and meta-analysis. Reprod. Biol. Endocrinol..

[72] Jamilian M., Samimi M., Ebrahimi F.A., Hashemi T., Taghizadeh M., Razavi M., Sanami M., Asemi Z. (2017). The effects of vitamin D and omega-3 fatty acid co-supplementation on glycemic control and lipid concentrations in patients with gestational diabetes. J. Clin. Lipidol..

[73] Johansen K.L., Kaysen G.A., Young B.S., Hung A.M., da Silva M., Chertow G.M. (2003). Longitudinal study of nutritional status, body composition, and physical function in hemodialysis patients. Am. J. Clin. Nutr..

[74] Norman K., Stobaus N., Zocher D., Bosy-Westphal A., Szramek A., Scheufele R., Smoliner C., Pirlich M. (2010). Cutoff percentiles of bioelectrical phase angle predict functionality, quality of life, and mortality in patients with cancer. Am. J. Clin. Nutr..

[75] Stobaus N., Pirlich M., Valentini L., Schulzke J.D., Norman K. (2012). Determinants of bioelectrical phase angle in disease. Br. J. Nutr..

[76] Norman K., Stobaus N., Pirlich M., Bosy-Westphal A. (2012). Bioelectrical phase angle and impedance vector analysis—Clinical relevance and applicability of impedance parameters. Clin. Nutr..

[77] Schwenk A., Beisenherz A., Romer K., Kremer G., Salzberger B., Elia M. (2000). Phase angle from bioelectrical impedance analysis remains an independent predictive marker in HIV-infected patients in the era of highly active antiretroviral treatment. Am. J. Clin. Nutr..

[78] Piccoli A., Rossi B., Pillon L., Bucciante G. (1994). A new method for monitoring body fluid variation by bioimpedance analysis: The RXc graph. Kidney Int..

[79] Chertow G.M., Lowrie E.G., Wilmore D.W., Gonzalez J., Lew N.L., Ling J., Leboff M.S., Gottlieb M.N., Huang W., Zebrowski B. (1995). Nutritional assessment with bioelectrical impedance analysis in maintenance hemodialysis patients. J. Am. Soc. Nephrol..

[80] Hoidrup S., Andreasen A.H., Osler M., Pedersen A.N., Jorgensen L.M., Jorgensen T., Schroll M., Heitmann B.L. (2002). Assessment of habitual energy and macronutrient intake in adults: Comparison of a seven day food record with a dietary history interview. Eur. J. Clin. Nutr..

